# Hand hygiene compliance and associated factors among health care providers in Gondar University Hospital, Gondar, North West Ethiopia

**DOI:** 10.1186/1471-2458-14-96

**Published:** 2014-01-30

**Authors:** Nura Muhammed Abdella, Mekuriaw A Tefera, Abebaw E Eredie, Timothy F Landers, Yewunetu D Malefia, Kefyalew Addis Alene

**Affiliations:** 1 Volentary and Counselling and Testing Center, College of Medicine and Health Sciences, Gondar University Hospital, University of Gondar, Gondar, Ethiopia; 2 Department of Environmental and Occupational Health and Safety, Institute of Public Health, College of Medicine and Health Sciences, University of Gondar, Gondar, Ethiopia; 3 Department of Nursing, College of Medicine and Health Sciences, University of Gondar, Gondar, Ethiopia; 4 The Ohio State University, Columbus, Ohio, USA; 5 Department of Epidemiology and Biostatistics, Institute of Public Health, College of Medicine and Health Sciences, University of Gondar, Gondar, Ethiopia

**Keywords:** Hand hygiene compliance, Health care provider, Gondar University Hospital

## Abstract

**Background:**

Health care associated infections are more predominant in developing countries where Hand hygiene compliance is associated with so many factors. However, these factors have not been studied so far in the study area. This study sought to determine Hand hygiene compliance and associated factors among health care providers.

**Methods:**

Institution based cross-sectional study was conducted from April to May, 2013 in Gondar University Hospital. Stratified sampling technique was used to select 405 health care providers. Standardized questionnaire and world health organization observational checklist was used to collect the data. Data was entered and analyzed by using SPSS version 20. Descriptive statistics and binary logistic regression model was used to summarize the result.

**Results:**

A total of 405 study participants were interviewed and observed with a response rate of 96.4%. Good Hand hygiene compliance of healthcare providers was found to be 16.5%. Having knowledge about hand hygiene compliance, (AOR = 3.80, 95% CI 1.60, 8.97), getting training (AOR = 2.60, 95% Cl 1.21, 5.62), the presence of individual towel/tissue paper (AOR = 1.91, 95% CI 1.03, 3.56) presence of alcohol based hand rub for Hand hygiene compliance (AOR = 6.58, 95% CI 2.67, 16.22) and knew the presence of infection prevention committees (AOR = 2.6, 95% CI 1.23, 5.37) were significantly associated with hand hygiene compliance.

**Conclusions:**

Hand hygiene compliance among health care providers in Gondar University Hospital was found to be low. It is better to give training on Hand hygiene compliance and provide Alcohol based hand rub and individual towel or tissue paper for hand hygiene compliance.

## Background

Hand hygiene (HH) is a compliance of cleansing hands using soap and water or using antiseptic hand rub for removal of transient microorganism from hands and in the way of keeping the skin condition. Any action of hand cleaning is referred to as hand hygiene [1,2]. Hand hygiene is the most simplest and effective measure to prevent infections. However, about 50% of health care associated infection occurs due to hand of health care providers (HCPs) [3].

Health care workers’ hands are the most usual type of vehicle for transmission of health care associated infections. Pathogenic micro organisms can stay for 2-60 minutes on health care workers’ hands. During patient care unless there is recommended hand hygiene compliance of health care providers kept, hands will be contaminated with microorganism [4]. It is estimated that annually about hundreds of millions of patients have suffered from health care associated infections (HCAIs) worldwide. The majority happened due to health care providers hands which will cause prolonged hospital stay, high amount of economical cost of patients, unnecessary laboratory investigation, high cost of drugs, and result to serious morbidity and mortality [5]. Therefore, good hand hygiene compliance is the simplest and the most valuable method of infection control in hospital [6]. Hospital acquired infection through the hands of health care workers is mostly due to poor hand hygiene of the health care providers [6]. Hand hygiene compliance is the way of minimizing the transmitting micro organism which may be multi drug resistant for those patients who have been infected and admitted in the hospital due to their cause and has got other infection by contaminated health care provider’s hands [7].

About 5%-10% of patients who are admitted in the hospital are at risk of getting infection. And health care associated infections are causes of death in the world. Large amount of patients life are at risk of getting infection [3].

“My five movement” for Hand hygiene compliance is considering the patient contact, patients’ surrounding and equipments during patient care and hand hygiene compliance for preventing health care associated infections. Those surroundings may have direct or indirect patient contact and those things which has frequent contact with health care providers [4].

Effective hand hygiene compliance s in hospitals play a key role in improving patient and provider safety, and in preventing the spread of health care-associated infections. However, Health care–acquired infections are rampant, with an estimated 1.7 million cases annually resulting in 99 000 deaths and significant added expenses [8]. Improper HH is one of the most important contributing factors to health care– acquired infections. Despite this information, hand hygiene compliance among health care workers in general is unacceptably low specially in developing countries like Ethiopia (range, 5%–89%; average, 38.7%) [9]. Therefore, the objective of this study is to assess the level of Hand hygiene compliance and associated factors among health care providers in Gondar University Hospital, Gondar, North West Ethiopia. The finding of this study could be useful evidence for scholars who are interested in this field.

## Methods

Facility based cross sectional study design supported by observation was employed to assess the level of Hand hygiene compliance and associated factors among health care providers.

The study was conducted at Gondar University Hospital [GUH] from April to May, 2013. This hospital is one of the oldest hospitals among medical schools in Ethiopia. It was established in 1954 as a public health college and training institute. It is located in North Gondar administrative zone, Amhara National Regional State, which is 738 km far from Addis Abeba, the capital city of Ethiopia. Currently Gondar town has one referral hospital, one private hospital and five government health centres. University of Gondar Hospital is a teaching Hospital which serves for more than five million people of the North Gondar zone and neighbouring regions. A total of 559 health care works are assigned in GUH to provide health services to the community. It has 129 physicians, 267 nurses, 33 midwives, 22 anaesthetists, 54 Lab technologists, 15 physiotherapists, 4 dental technologists, 8 radiographer, 15 Optometry, 12 health officers. The hospital has different departments and 500 beds for admitted patients.

The study population was all health care providers who are working at Gondar University Hospital. Those health care providers who were on leave during the data collection period were excluded.

The sample size (n) was computed by single population proportion formula [n = [(zα/2)^2^ * P (1-P)]/d^2^] by assuming 95% confidence level of Z α/_2_ = 1.96, margin of error 5% and to have maximum sample size we have taken 50% proportion. By considering this the calculated sample was 384. With adjustment for non response (10%) the sample size became 423.

Stratified sampling technique was employed in order to select a representative sample of HCPs from each disciplinary team. First HCPs in Gondar university hospital (GUH) was stratified by their profession for HHC as nurses, midwifes, physicians, health officers, anesthetises, physiotherapist, laboratory technologist, X-ray technologist and optometrist. Then finally proportional number of participants (health care providers) was selected by simple random sampling technique using lottery method from the list of health care providers in each stratum. There were 559 lists of HCPs in their respective working disciplines in the hospital.

The primary dependent variable was Hand hygiene compliance and other independent variables were defined as a categorical variable with the following:

***Good hand hygiene compliance: -*** health care providers who scored ≥ 50% of the observational checklists [10,11].

***Poor hand hygiene compliance: *-** health care providers who scored < 50% of the observational checklists [10,11].

***Knowledgeable: *-** health care providers who scored the mean and above the mean value of the knowledge questions.

***Not knowledgeable: -*** health care providers who scored below the mean of the knowledge questions value.

***Patient surrounding: -*** the space where patient lied and its surrounding can be touched by the patients and HCPs at any time whenever giving a care of a patient.

***Health Care Providers: -*** health professionals who provide care and have a direct contact to the patients (physicians, nurses, health officer, physiotherapists’, laboratory technologist, Optometry, anesthetists and radiographer).

***Patient contact: -*** HCPs who involve in touching patients while examining and giving care.

To ensure quality of data, standardized checklist and structured questionnaires was used. Pre-test was done on 25 health care workers out of the study area and necessary correction was done accordingly. Intensive training was given to data collectors and supervisor for one day on how to approach study subjects, on how to use the questionnaire, the observational checklists and how to do concealed observation. Supervision was done at the spot by investigator and supervisors. The collected data was checked for the completeness, accuracy and clarity by the investigator and supervisors. Appropriate measure was taken on time for completeness before data entry. Data clean up and cross-checking was done before analysis.

Each completed questionnaire was checked visually for completeness before fed to computer. The data was entered and analyzed using SPSS version 20. Descriptive statistics like frequencies and cross tabulation was performed. Crude and adjusted odds ratios with 95% confidence interval was used to determine the strength of association between dependent and independent variables. Variables having P-value ≤ 0.05 was considered as significant.

Ethical clearance was obtained from Institutional Review Board of University of Gondar. Then official letter obtained from administrative body of Gondar university hospital. The purpose of study was well explained to the study participants and informed consent was obtained. Confidentiality was maintained at all levels of the study by avoiding use of name and other identifiers. Participants’ involvement in the study was on voluntary basis; participants who were unwilling to participate in the study and those who wish to quit their participation were informed to do so without any restriction.

## Results

A total of 405 study participants were interviewed with self administered questions and observed by observational checklist with the response rate of 96.4%. The mean age (±SD) of respondents was 28.33 ± 5.4 years. Majority of the respondents 82% were Orthodox Christians. About half (48.4%) and one fourth (22.2%) of the respondents were nurse and physicians respectively. Two hundred fourteen (52.8%) reported to be single. Four point four (4.4) years were the Mean working years (Table 1).

**Table 1:** Socio-demographic characteristic of the health care providers in Gondar University Hospital [GUH], Northwest Ethiopia, 2013 (n=405)

**Variables**		**Frequency**	**Percent (%)**
Age	18-24	77	19
	25-34	283	69.9
	≥35	45	11.1
Sex	Male	270	66.7
	female	135	33.3
Religion	Orthodox	332	82.0
	Muslim	45	11.1
	Protestant	21	5.1
	others	7	1.7
Profession	Physician	90	22.2
	Nurse	196	48.4
	Lab-technologist	41	10.1
	HO	9	2.2
	Physiotherapist	11	2.7
	Midwives	25	6.2
	Anesthetists	15	3.7
	Others HCPs	18	4.4
Educational level	Diploma	55	13.6
	BSc (1^st^ degree)	285	70.4
	2^nd^ degree and above	65	16
Marital status	Married	183	45
	Single	214	52.8
	Divorced	5	1.2
	Widowed	1	0.25
	Separated	2	0.5
Year of working experience	<1 year	62	15.3
	1-4 years	243	60.0
	>4 years	100	24.7

**Figure 1:**
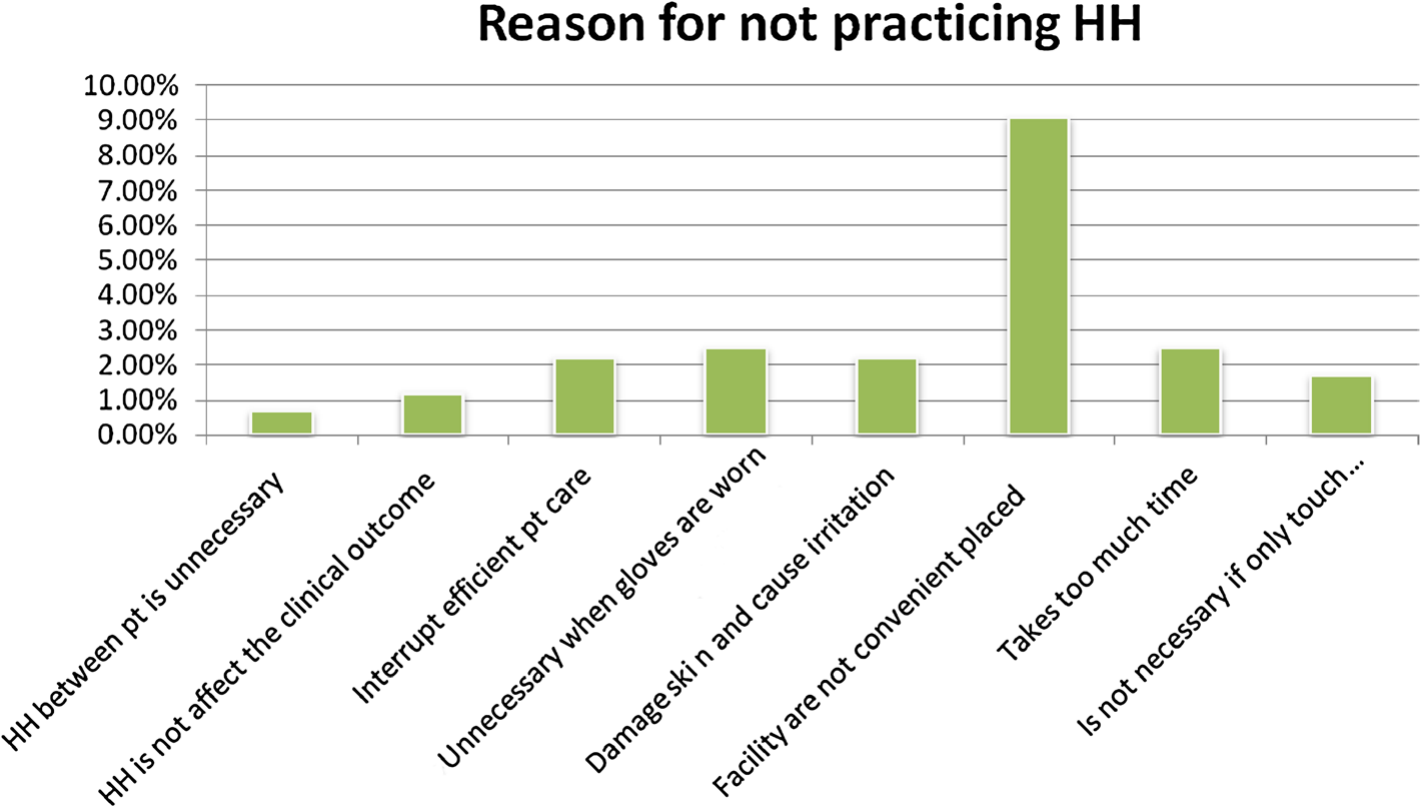
Respondents’ reasons for not practicing hand hygiene in Gondar University Hospital [GUH], Gondar, North West Ethiopia, 2013.

Majority 312 (77.3%) of the respondents were knowledgeable on Hand hygiene compliance [HHC]. Two hundred forty four (60.2%) HCPs had trained for HHP. Two hundred thirty one (57%) assured the presence of Alcohol Based Hand Rub [ABHR]. One hundred forty eight (36.5%) reported that the presence of individual towel or tissue paper for drying in their working area. Regarding knowledge on the presence of infection prevention [IP] committees, about two hundred twenty six (55.8%), of the respondents knew the presence IP committees (Table 2). Not practicing hand hygiene [HH] was asked by self administered questionnaires. About 37(9.1%) of the respondents reason out that facility was not conveniently placed followed by Unnecessary when gloves are worn (Figure 1).

**Table 2:** **Variables related to hand hygiene practice in Gondar University Hospital [GUH**]**, Gondar, Northwest Ethiopia, 2013 (n=405)**

**Variable**	**Frequency**		**Percent**
Knowledge	Yes	313	77.3
	No	92	23.7
Frequently keep HH	Yes	344	84.9
	No	61	15.1
Taking training	Yes	244	60.2
	No	161	38.8
Hospital promote the importance of HH to the staffs	Yes	285	70.4
	No	120	29.6
The availability of soap and water	Yes	164	40.5
	No	241	59.5
The availability of hand washing sink	Yes	173	42.7
	No	232	57.3
The availability of individual towel or tissue paper for drying	Yes	148	36.5
	No	257	63.5
The availability of wall mount/ individual ABHR	Yes	231	57
	No	174	43
The availability of gloves	Yes	267	65.9
	No	138	34.1
Knew the presence of IP committees	Yes	226	55.8
	No	179	44.2

In the Bivariate analysis knowledge of HHC, taking training on HH, the hospital promoting the importance of HH compliance, the availability of hand washing sink, soup and water, individual towel/tissue paper for drying, the availability of ABHR for HH compliance, knew the presence of IP committees are significantly associated with Hand hygiene compliance (Table 3).

**Table 3:** Factors associated with hand hygiene compliance among health care providers in Gondar University Hospital [GUH], Gondar, Northwest Ethiopia, 2013 (n=405)

**Variables**		**HH practice**		**COR (CI 95%)**	**AOR (CI 95%)**
		**Yes**	**No**		
Age	18-24	13	64	r	
	25-34	44	239	0.91 (.46, 1.78)	
	≥35	10	35	1.41 (.56, 3.54)	
Sex	Male	47	223	r	
	Female	20	115	0.83 (0.47, 1.46)	
Religion	Orthodox	52	280	r	
	Muslim	9	36	1.35 (0.61, 2.96)	
	Others*	6	22	1.47 (.57, 3.80)	
Profession	Physician	11	79	r	
	Nurse	43	178	1.74 (.85, 3.54)	
	Lab-tech	8	33	1.74 (.64, 4.72)	
	Other HCPs**	5	48	0.75 (0.25, 2.28)	
Educational level	Diploma	12	43	r	
	BSc (1^st^ degree)	45	240	0.67 (.33, 1.37)	
	2^nd^ degree & above***	10	55	0.65 (.26, 1.65)	
Marital status	Married	33	152	1.19 (.70, 2.01)	
	Unmarried****	34	186	r	
Year of working exp	< 1 year	11	51	r	
	1-4 years	36	207	0.81 (.38, 1.69)	
	>4 years	20	80	1.16 (.51,2.62)	
Knowledge of HHP	Yes	60	253	2.88 (1.27, 6.54)	3.80 (1.60, 8.97)
	No	7	85	r	
taking training on HH	Yes	57	187	4.60 (2.27, 9.32)	2.60 (1.21, 5.62)
	No	10	151	r	
Hospital promoting the importance of HHP	Yes	61	224	5.17 (2.17, 12.33)	
	No	6	114	r	
Availability of soap and water in working ward	Yes	43	121	3.20 (1.86,5.55)	
	No	24	217	r	
Availability of sink in working ward	Yes	41	132	2.46 (1.44,4.21)	
	No	26	206	r	
Availability of Ind towel/tissue paper	Yes	42	106	3.70 (2.13,6.35)	1.90 (1.02, 3.53)
	No	25	232	r	
Availability of ABHR in the ward	Yes	61	170	10.01 (4.23, 23.87)	6.58 (2.67, 16.22)
	No	6	168	r	
Availability of glove in the ward	Yes	40	227	0.72 (.42, 1.24)	
	No	27	111	r	
Knew presence of IP committees	Yes	56	170	5.00 (2.55,9.94)	2.60 (1.23, 5.37)
	No	11	168	r	

*protestant and others, **HO, dental technologists, radiographers, physiotherapist, Anesthetists, ***masters, specialists Dr, resident Dr, ****reference Divorced, widowed, r-reference.

The multivariate analysis was used to identify factors that were predictive of hand hygiene compliance. Knowledge of HHC, taking training on HH, availability of individual towel/tissue paper, availability of ABHR in the ward and knew presence of IP committees are independently associated factors with hand hygiene compliance (Table 3).

## Discussion

Poor HH compliance of HCPs and its complication of HCAIs have impact on the patients, visitors and health care providers. Several factors which may be the health care providers’ and the health care systems related to poor HH compliance of HCPs [12]. This study tried to assess Hand hygiene compliance of HCPs and the associated factors.

Good Hand hygiene compliance of health care providers as measured by this study was found to be 16.5%. This finding is slightly above with study conducted in Africa Ghana teaching hospital which showed that the overall HH compliance was 12% [10]. But this finding was lower than other studies done in Asia countries by using similar method, Kuwait and India in which HHC was 33.4% and 43.4% respectively [13,14]. This might be due to lack of hand hygiene resources in our countries specifically in Gondar University Hospital and there may be lack of knowledge about HH compliance. Another reason might be the study performed as early as the new method of HH compliance using ABHR has been introduced in Gondar University Hospital, Even though it is lower than 50% WHO recommended HH compliance measurement method.

Knowledge to HH compliance was found to be associated with HH compliance. Those who had Good knowledge on HH had 3.8 times more compliance than poor Knowledge. This was in line with other similar study done in Kuwait which showed that knowledge of HCPs were significantly associated with good HH compliance [14]. This study also gives additional evidence of knowledge on HHC will help to compliance HH with recommended way, and knowledge of HHC will help to identify the advantage and disadvantage of HHC and Knowledge will help to identify the way of HCAIs transmission and how it is prevented.

Training about HH compliance was found to be significantly associated with HH compliance of health care providers. Those who were trained had 2.6 times more compliance than those who were not trained. Which was also supported by money other studies done in India, UK and China which showed that training had positive relationship with HH compliance in all medical staffs [15-17]. This might be due to the fact that training built the capacity of health care providers which had a significant association in HH compliance. The other reason might be those HCPs who had got training are expected to be a role models for others in terms of practicing good HH. Training might be very vital to remind HHP. Post training follow up might contribute for better HHP.

The presence of ABHR was positively associated with HH compliance in which those who had access for ABHR in their ward had 6.5 times more likely to compliance than those who had not access on ABHR. This is in line with other studies done in Taiwan and Brazil. The availability of ABHR resulted in significant improvement HH compliance of HCPs [18,19]. This might be the presence of ABHR at point of care will remind the HCPs to do HH, ABHR might be easy for implementing HH.

The presence of individual towel/tissue paper in the working area was positively associated with HHP. Those who had access for individual towel/tissue paper for drying in their ward is 2 times more likely compliance HH than who had not access. Which is in line with study done in Australia, the presence of hand drier will improve HHC [20]. This might be the health care providers frightened that the wet hand will more contaminates than dry hand. Another reason might be inaccessibility or unavailability of towel/tissue paper make hand wet for long time and hindered practicing HH.

The presence of IP committees was positively associated with HH compliance of HCPs. Those health care providers who know the presence of IP committee are about 2.6 times more likely to compliance than those who didn’t know the presence of IP committee. This is in line with the studies done in Italy and Ontario explained that the presence of IP committees will result in the reduction of HCAIs [21,22]. This may be explained by IP committees may provides supervision on the HH compliance of the health care providers, and audit of HH compliance and may providers to the hospital to address the gap, and possible the IP committees may give feedback to HCPs at the point of care. Those who know the presence of IP committee may contact the committee and get necessary materials for HHP. It might be also due to the fact that those who know the presence of IP committee may have a worry of that they may be supervised by the committee and criticized if they were not doing HHP.

The study was conducted with a certain limitation: due to the cross-sectional nature of this study temporal relationship couldn’t be established between the explanatory and outcome variable. Even if we have tried to control the hawthorn’s effect, to some extent this study was subjected to this bias.

## Conclusion

Hand hygiene complianceamong HCPs in Gondar University Hospital was found to be low. Good knowledge of HHC, taking training, the presence of ABHR in working area, the presence of individual towel or tissue paper in working area and Knew the presence of IP committees were found to be the independent predictors for HHC in Gondar University Hospitals. It is better to give training on hand hygiene and provide necessary material like alcohol based hand rub and individual towel or tissue paper.

## Competing interests

The authors declare that they have no competing interests.

## Authors’ contributions

NM wrote the proposal of this research, KA, AE, TFL, YD and MA revised the proposal and incorporate some comments. NM, KA, AE, TFL, YD and MA participated in the literature review. NM, KA, YD and MA participated in data collection and analysis. NM, YD and MA wrote the final manuscript. All authors read and approved the final manuscript.

## Pre-publication history

The pre-publication history for this paper can be accessed here:

http://www.biomedcentral.com/1471-2458/14/96/prepub

## References

[B1] Pittet D Improving adherence to hand hygiene compliance: a multidisciplinary approach Emerg Infect Dis 2001 7 2 234 240 10.3201/eid0702.010217 11294714 PMC2631736

[B2] MOH Best compliance s for hand hygiene in all health care settings 2009 Ontario, Canada: MOH

[B3] Martin-Madrazo C Canada-Dorado A Salinero-Fort MA Abanades-Herranz JC Arnal-Selfa R Garcia-Ferradal I Espejo-Matorral F Carrillo-de Santa-Pau E Soto-Diaz S Effectiveness of a training programme to improve hand hygiene compliance in primary healthcare BMC Public Health 2009 9 469 10.1186/1471-2458-9-469 20015368 PMC2806875

[B4] WHO WHO Guidelines on hand hygiene in health care: a summary First Global Patient Safety Challenge Clean Care is Safer Care 2009 Geneva: World Health Organization 23805438

[B5] Mathai E Allegranzi B Kilpatrick C Pittet D Prevention and control of health care-associated infections through improved hand hygiene Indian J Med Microbiol 2010 28 2 100 106 10.4103/0255-0857.62483 20404452

[B6] CDC How-to Guide: improving hand hygiene Institute health care improvement 2003 USA: CDC 217 223

[B7] Monnet DL Sprenger M Hand hygiene compliance s in healthcare: measure and improve Euro Surveill 2012 17 18 20166 22587954 10.2807/ese.17.18.20166-en

[B8] Sax H Allegranzi B Uckay I ‘My five moments for hand hygiene’: a user-centered design approach to understand, train, monitor and report hand hygiene J Hosp Infect 2007 67 1 9 21 10.1016/j.jhin.2007.06.004 17719685

[B9] World Health Organization WHO guidelines on hand hygiene in health care 2009 Geneva: WHO Press

[B10] Owusu-Ofori A Jennings R Burgess J Prasad PA Acheampong F Coffin SE Assessing hand hygiene resources and compliance s at a large african teaching hospital Infect Control Hosp Epidemiol 2010 31 8 802 808 10.1086/654005 20569112

[B11] Novoa MA Pi-Sunyer T Sala M Molins E Castells X Evaluation of hand hygiene adherence in a tertiary hospital Am J Infect Control 2007 35 676 683 10.1016/j.ajic.2007.03.007 18063133

[B12] Erasmus V Brouwer W van Beeck EF Oenema A Daha TJ Richardus JH Vos MC Brug J A qualitative exploration of reasons for poor hand hygiene among hospital workers: lack of positive role models and of convincing evidence that hand hygiene prevents cross-infection Infect Control Hosp Epidemiol 2009 30 415 419 10.1086/596773 19344264

[B13] Al-Wazzan B Salmeen Y Al-Amiri E Abul A Bouhaimed M Al-Taiar A Hand hygiene compliance s among nursing staff in public secondary care hospitals in Kuwait: self-report and direct observation Med Princ Compliance 2011 20 4 326 331 10.1159/000324545 21576991

[B14] Sharma S Sharma S Puri S Whig J Hand hygiene compliance in the intensive care units of a tertiary care hospital Indian J Community Med 2011 36 3 217 221 10.4103/0970-0218.86524 22090677 PMC3214448

[B15] Lam BC Lee J Lau YL Hand hygiene compliances in a neonatal intensive care unit: a multimodal intervention and impact on nosocomial infection Pediatrics 2004 114 5 e565 e571 10.1542/peds.2004-1107 15492360

[B16] Suchitra JB LaKshmi D Impact of education on knowledge, attitudes and compliance s among various categories of health care workers on nosocomial infections Indian J Med Microbiol 2007 27 3 181 187 17901633 10.4103/0255-0857.34757

[B17] Randle J Clarke M Storr J Hand hygiene compliance in healthcare workers J Hosp Infect 2006 64 205 209 10.1016/j.jhin.2006.06.008 16893593

[B18] Chen YC Sheng WH Wang JT Chang SC Lin HC Tien KL Hsu LY Tsai KS Effectiveness and limitations of hand hygiene promotion on decreasing healthcare-associated infections PLoS One 2011 6 11 e27163 10.1371/journal.pone.0027163 22110610 PMC3217962

[B19] Santana LS Furtado CHG Coutinho PA Medeiros SAE Assessment of healthcare professionals’ adherence to hand hygiene after alcohol-based hand rub introduction at an intensive care unit in Sa˜o Paulo, Brazil Infect Control Hosp Epidemiol 2007 28 3 365 367 10.1086/510791 17326033

[B20] Huang C Ma W Stack S The hygienic efficacy of different hand-drying methods: a review of the evidence Mayo Clin Proc 2012 87 8 791 798 10.1016/j.mayocp.2012.02.019 22656243 PMC3538484

[B21] Sydnor ERM Perl TM Hospital epidemiology and infection control in acute-care settings Hosp Epidemiol Acute Care Settings 2011 24 1 141 173 10.1128/CMR.00027-10 PMC3021207 21233510

[B22] Vearncombe M Card LM Cividino M Freeman R Gardam M Best compliance s for infection prevention and control programs in Ontario Best Compliance s for Infection Prevention and Control Programs in Ontario 2008 Toronto, Canada: Ontario Ministry of Health and Long-Term Care

